# Unveiling *Leptospira* prevalence and exposure in sanitation workers, a cross-sectional study in Ningbo City, China

**DOI:** 10.3389/fpubh.2025.1627155

**Published:** 2025-07-02

**Authors:** Hui Su, Keye Xu, Wuke Wang, Yuhui Liu, Guohua Ping, Dingyi Bo

**Affiliations:** ^1^Department of General Surgery, Ningbo No.2 Hospital, Ningbo, Zhejiang, China; ^2^Department of Microbiology, Ningbo Municipal Center for Disease Control and Prevention, Ningbo, Zhejiang, China; ^3^Department of Quality Control, Ningbo Municipal Center for Disease Control and Prevention, Ningbo, Zhejiang, China; ^4^Department of Disease Control, Haishu District Center for Disease Control and Prevention, Ningbo, Zhejiang, China

**Keywords:** sanitation workers, *Leptospira*, leptospirosis, carriers, colonization

## Abstract

**Background:**

The environmental presence of pathogenic *Leptospira* species poses a substantial threat to human health. Sanitation workers, due to their frequent exposure to contaminated soil or water, are at an increased risk of infection. This study aimed to better understand the risk factor of leptospirosis, pathogen exposure, and carriage among sanitation workers in Ningbo, so to help prevent and manage future outbreaks.

**Methods:**

A total of 306 samples were collected, comprising 102 whole blood samples, 102 serum samples, and 102 urine samples from sanitation workers which were categorized into 3 groups. Serum samples were analyzed using ELISA to detect IgM and IgG antibodies. qPCR targeting *lipL32* and *sec Y* were employed on urine and whole blood samples. PCR were performed targeting *sec Y* followed by sequencing using Sanger method, alignment using DNASTAR MegAlign and MEGA X. Tested positive bio-samples subsequently cultured in EMJH broth supplemented with 5-fluorouracil to facilitate bacterial growth, and examined using a dark-field microscope.

**Results:**

The questionnaires results showed long working hours (most frequently reported risk factor 60.78%) and employment in garbage sorting (52.94%) were associated with elevated risk of infection (OR = 1.92 and OR = 1.68; *p* = 0.004 and *p* = 0.03, respectively), while use of boots (56.86%), masks (41.18%) and soaps (92.16%) can reduce the risk (OR = 0.33, OR = 0.55 and OR = 0.43; *p*<0.001, *p* = 0.03 and *p* = 0.007, respectively). 59.8% of participants tested positive for IgM. qPCR analysis targeting the *sec Y* gene revealed a positivity rate of 32.4% in blood samples and 7.8% in urine samples. Notably, the *lipL32* gene was not detected in any samples. 42 *secY* gene amplicons obtained from whole blood samples and 8 from urine samples exhibited high sequence similarity to *Leptospira interrogans* (*L. interrogans*) when analyzed using DNASTAR MegAlign, clustering with *L. interrogans* (GenBank accession number OM456545.1). However, culture results showed negative through 2 months observation.

**Conclusion:**

This observation indicates the prevalence of a pathogenic *L. interrogans* subtype, which lacks the *lipL32* gene, among asymptomatic sanitation workers. It is imperative for these workers to possess knowledge about infection risks and preventive measures to mitigate the likelihood of infection.

## 1 Introduction

Leptospirosis, a zoonotic disease prevalent worldwide, is among the most widespread endemic diseases in tropical and subtropical regions (1, 2). It is recognized for its epidemic potential and poses a significant public health risk. Despite its global presence, leptospirosis remains a neglected disease in many areas. The causative agent, *Leptospira* species, is a motile spirochete which categorized into pathogenic, intermediate, and saprophytic (non-pathogenic) groups based on pathogenicity. Pathogenic *Leptospira* species are currently classified: *L. alexanderi, L. alstonii, L. borgpetersenii, L. interrogans (sensu stricto), L. kirschneri, L. kmetyi, L. mayottensis, L. noguchii, L. santarosai* and *L. weili* (3). Intermediate *Leptospira are* positioned between pathogenic and non-pathogenic types, genetically closer to pathogenic *Leptospira* and retain certain pathogenicity-related genes (4). Saprophytic *Leptospira*, like *Leptospira biflexa*, are non-pathogenic but crucial in ecosystems (5). The clinical manifestations of this disease range from mild symptoms to a flu-like syndrome, and may progress to include jaundice, pulmonary hemorrhage, or even result in death. Some outbreaks occurred in different areas and countries showed that the disease has become re-emerging. Recent studies have highlighted the emergence of new serovars and changing epidemiological patterns, which complicate control efforts (6–8). The morbidity rate of leptospirosis in Indonesia is estimated to be 39.2 per 100,000 individuals, which is higher than in other Asian countries, such as India (19.69 per 100,000) and the Philippines (14.98 per 100,000) (9). This disease has re-emerged due to outbreaks following natural disasters (10–12).

The environment serves as a reservoir for *Leptospira* infection. *Leptospira* can persist in soil and aquatic environments for extended periods under conditions of high precipitation and warmth, which are conducive to its proliferation (13–16). The widespread presence of *Leptospira* species is likely due to their ability to adapt genetically and survive in various environments, as shown by the flexible nature of their genome (17). Transmission of pathogenic *Leptospira* strains to humans occurs primarily through contact with environments contaminated by the urine of infected animals or reservoir hosts (18, 19). Rodents are recognized as maintenance hosts as the bacteria can asymptomatically colonize their kidneys and be excreted into the environment via urine (20). Studies identified the presence of long-term leptospiruria excreted by asymptomatic and seronegative individuals in endemic regions (20, 21). Another study reported that 20% of the population in leptospirosis-endemic areas exhibited leptospiruria without any preceding symptoms or fever (22). These findings suggest that *Leptospira* may adapt to and persist in the human kidney for extended periods (years) in without manifesting symptoms. Repeated exposure to *Leptospira* may facilitate the bacterium’s adaptation to the host immune system.

LipL32 protein as virulence marker (23) is crucial for diagnostic tests due to its structural properties and the high conservation of its gene among pathogenic *Leptospira* strains. Real-time PCR assays targeting *lipL32* enable rapid and specific detection of pathogenic *Leptospira* in clinical samples, aiding in the timely diagnosis of leptospirosis (24). LipL32 binds calcium ions, enhancing its stability and interaction with host proteins, highlighting its importance in bacterial pathogenesis (25, 26). The *secY* gene is vital for detecting and diagnosing leptospirosis, acting as a dependable phylogenetic marker due to its conserved yet variable sequence (27). It is commonly used to broadly identify *Leptospira* strains, aiding in the study of the disease’s epidemiology and transmission. Although not a virulence factor, SecY indirectly supports pathogenicity by facilitating the secretion of proteins linked to virulence.

Ningbo, a coastal city in Zhejiang province, is situated in a temperate zone that is susceptible to flooding due to its high rainfall season and the confluence of three rivers. These conditions contribute to Ningbo may being an endemic area for leptospirosis. Sanitary workers and related work including drivers who are responsible for garbage transfer and operators who deal with garbage sorting may frequently in contact with the city’s environment, are at increased risk of infection. The primary risk factors for *Leptospira* infection include frequent exposure to contaminated environments, especially when personal protective equipment is not utilized during work (28). Occupational exposure and seasonal factors play a crucial role in the transmission dynamics of leptospirosis in the region. A study in Wenzhou, Zhejiang Province conducted from 2020 to 2022 reported 41 cases of human leptospirosis, with a significant proportion of cases occurring among males and individuals engaged in farming (29). We decided to carry out a study to better understand the risk factor of leptospirosis, pathogen exposure, and carriage among sanitary workers, drivers, and operators in the Ningbo area, so to help prevent and manage future outbreaks.

## 2 Methods

### 2.1 Sample collection and questionnaire

Samples were collected from the urine, serum, and whole blood of 102 sanitation workers including 91 (89.22%) males and 11 (10.78%) females at the Environmental Sanitary Management Center which in charge of waste disposal in Haishu district, located in the western part of Ningbo City, Zhejiang Province. This district was selected due to its flood-prone characteristics and the significant presence of pathogenic and saprophytic *Leptospira* in the soil of farmland within Haishu district (data not published). Our participants were divided into three groups: drivers group, operators group and sanitary workers group. The drivers were primarily responsible for collecting trash and garbage from various communities and transporting it to transfer stations or landfill sites. The operators, all of whom were based at transfer sites, were tasked with managing the waste sorting. Sanitary workers were responsible for street cleaning and emptying public dustbins. We used a yes/no questionnaire to assess if personal protective equipment (PPE) was properly worn. Those with no contact with potentially contaminated environments (like water, gutters, or waste) were excluded.

### 2.2 Molecular identification

Urine (300 μL) and blood samples (350 μL) were processed with the Qiacube HT system and IndiSpin® QIAcube® HT Pathogen Kit (Qiagen, Germany) as per the manufacturer’s instructions. Extracted DNA underwent qPCR for *secY* using the *Leptospira* real-time PCR kit (MABSKY, Shenzhen, China) and the *lipL32* gene (primers laboratory owned). An internal control plasmid confirmed no *secY* qPCR inhibition in singleplex reactions. *lipL32*-45F (5’-AAGCATTACCGCTTGTGGTG-3′) and *lipL32*-286R (5’-GAACTCCCATTTCAGCGATT-3′) were used to amplify a 242 bp fragment, detected by the probe *lipL32*-189P (FAM-5’-AAAGCCAGGACAAGCGCCG-3’-BHQ1) (24, 30). To check for PCR inhibitors and monitor nucleic acid extraction, samples were tested for the housekeeping gene *RNAseP* (Genbank accession no. NM_066413). The forward primer of *RNAseP*3F (5’-CCAAGTGTG AGGGCTGAAAAG-3′) and *RNAseP*3R (5’-TGTTGTGGCTGATGA ACTATAAAAGG-3′) were selected to amplify a fragment detected by the probe *RNAseP*3 (FAM-5’-CCCCAGTCTCTGTCAGCACTCCC TTC-3’-BHQ1) (30). Samples were positive if the cycle threshold (*Ct*) value was under 40. Non-template controls were randomly included to ensure no contamination, with all negative controls confirming negative results. Final reaction conditions for ABI Q5 were 10 μL 2 × AceQ universal U^+^ probe Master Mix (Vazyme, Nanjing, China), 0.4 μL for each primer (10 μM), 0.2 μL Taqman probe (10 μM) and 5 μL template DNA extract from urine and blood specimens in a final volume of 20 μL. The amplification protocol consisted of 5 min at 95°C, followed by 45 cycles of amplification (95°C for 10 s and 60°C for 30 s).

### 2.3 Phylogenetic analysis

A conventional PCR targeting the *secY* gene was performed using Q5® High-Fidelity 2X Master Mix (NEB, USA) with specified primers. The reaction mix included 5 μL DNA template, 12.5 μL Master Mix, and 1.25 μL of each primer, totaling 25 μL. Primers were as followed: forward primer 5’-CTGAATCGCTCGTATAAAAGT-3′ and reverse primer 5’-GGAAAACAAATGGTCGGAA-3′ (National Standards WS290-2008). The PCR conditions comprised an initial denaturation at 98°C for 30 s, followed by 35 cycles of denaturation at 98°C for 10 s, annealing at 55°C for 30 s, and extension at 72°C for 30 s, concluding with a final extension at 72°C for 2 min. The PCR product was subjected to electrophoresis on a 1% agarose gel, followed by purification and sequencing using the Sanger method at Tsingke Biotech (Hangzhou, Zhejiang). The resulting DNA sequence underwent homology analysis using DNASTAR MegAlign and MEGA X. The *secY* gene sequence identified in the newly sequenced organism was compared with those from the genus *Leptospira*, obtained from GenBank. Sequence alignment was performed using CLUSTAL W. Phylogenetic analysis was conducted with MEGA X, employing the Neighbor-Joining method to estimate the distances between aligned sequences.

### 2.4 Serological examination

Serum samples were tested for IgM and IgG using a *leptospira* ELISA kit (31, 32) (NovaTec, Germany). Microtiter plates coated with specific antigens capture antibodies from the samples. After washing away unbound material, an HRP-labeled conjugate was added to bind the captured antibodies, followed by a second wash to remove unbound conjugate. The immune complex with the conjugate is visualized using Tetramethylbenzidine (TMB), producing a blue product whose intensity reflects the antibody levels in the sample. Sulphuric acid stops the reaction, turning it yellow. Absorbance is measured at 450/620 nm with an ELISA Microtiterplate reader.

### 2.5 Bacteria culture

Urine and blood samples of 100 μL were introduced into 10 mL of Ellinghausen–McCullough–Johnson–Harris (EMJH) broth supplemented with 5-fluorouracil (5-FU). *Leptospira* is known to be resistant to 5-FU, and its inclusion in the medium effectively inhibits non-*Leptospira* bacteria (33). These samples were incubated at 28°C for a duration of 2 months. Weekly observations were conducted using a dark-field microscope to monitor for bacterial growth.

### 2.6 Statistics analysis

Data analysis was conducted utilizing Python version 3.9. Descriptive statistics were employed to summarize the characteristics of the participants. Bivariate associations between leptospirosis infection and potential predictors were evaluated using chi-square or Fisher’s exact tests. Variables that demonstrated *p* < 0.20 in the bivariate analysis were incorporated into a multivariable logistic regression model to estimate adjusted odds ratios (aOR) and 95% confidence intervals (CI).

Formula: $\log(\frac{P(Y=1)}{1-P(Y=1)})=\beta 0+\beta_{1}X_{1}+\beta_{2}X_{2}+\ldots +\beta_{k}X_{k}$

Y = leptospirosis infection, X_1_ to X_10_ represent the independent variables. The model’s fit was assessed using the Hosmer-Lemeshow test and the area under the receiver operating characteristic curve (ROC-AUC). *p* < 0.05 was considered indicative of statistical significance. The phylogenetic tree was generated together with MEGA X software and ChiPlot^1^ employing 1,000 bootstrap replicates.

## 3 Results

### 3.1 Demographic findings

The questions included in the questionnaires are detailed in Table 1. The most frequently reported risk factors were long working hours (60.78% affirmative responses) and employment in garbage disposal (52.94% affirmative responses). In contrast, working in flood-affected areas was perceived as a relatively lower risk, with 35.29% affirmative responses. The practice of cleaning the body with soap was nearly universal, with 92.16% affirmative responses, followed by the use of boots while working, at 56.86%. Conversely, cleaning the body with only water and using masks while working were the least adopted practices, each with 41.18% affirmative responses. Long working hours and employment in garbage disposal were associated with a significantly elevated risk of infection (OR = 1.92 and OR = 1.38; *p* = 0.004 and *p* = 0.03, respectively). The use of boots, masks, and cleaning the body with soap were associated with reductions in infection risk by 67, 45, and 57%, respectively, as detailed in Table 2.

**Table 1:** List of leptospirosis risk factors questions.

Risk and prevention	Questions	Yes (%)	No (%)
Risk of infection	Wound in foot	37.25	62.75
	Wound in hand	46.08	53.92
	Long working hours	60.78	39.22
	Working at flood area	35.29	64.71
	Working at garbage disposal	52.94	47.06
Prevention efforts	Using gloves while working	49.02	50.98
	Using mask while working	41.18	58.82
	Using boots while working	56.86	43.14
	Cleaning body using soap	92.16	7.84
	Cleaning body using only water	41.18	58.82

**Table 2:** Logistic regression analysis of risk factors and protective measures for *Leptospira* infection.

Variables	*β*	OR	95% CI	*p* value
Wound in foot	0.25	1.28	[0.75, 2.20]	0.37
Wound in hand	0.18	1.20	[0.70, 2.05]	0.51
Long hours working	0.65	1.92	[1.22, 3.02]	0.004
Working at flood area	0.10	1.11	[0.62, 1.97]	0.73
Garbage disposal	0.52	1.68	[1.05, 2.70]	0.03
Using gloves	−0.20	0.82	[0.50, 1.34]	0.43
Using mask	−0.60	0.55	[0.32, 0.94]	0.03
Using boots	−1.10	0.33	[0.18, 0.61]	<0.001
Cleaning with soap	−0.85	0.43	[0.23, 0.79]	0.007
Cleaning with Water only	0.15	1.16	[0.68, 1.99]	0.59

### 3.2 PCR and qPCR results

The cohort consisted of 91 male and 11 female participants. Assays for the *lipL32* gene yielded no positive results. However, qPCR analysis of the *sec Y* gene showed positive results in 33 (32.4%) whole blood samples and 8 (7.8%) urine samples, with 31 blood and 8 urine samples from males and 2 blood samples from females testing positive (Supplementary Table 1). Positivity rates for males (34.1%; 44.0%) and operators (45.5%; 59.1%) based on blood samples detected by qPCR and PCR were higher compared to females and other occupational groups. Both methods demonstrated similar sensitivity for urine samples. The *secY* gene, with a 285 bp fragment, was identified in 42 whole blood samples and 8 urine samples (Supplementary Tables 1, 2). Conventional PCR appeared more sensitive than qPCR, detecting 9 additional samples. Only 27 blood samples can be detected by both methods (Table 3).

**Table 3:** Display of positive numbers from blood and urine samples by PCR and qPCR.

Gene	Method	Positive numbers (whole blood)	Positive numbers (urine)
*lipL32*	qPCR	0	0
*secY*	qPCR	33	8
*secY*	PCR	42	8
*secY*	qPCR+PCR	27	8

### 3.3 Phylogenetic tree

**Figure 1:**
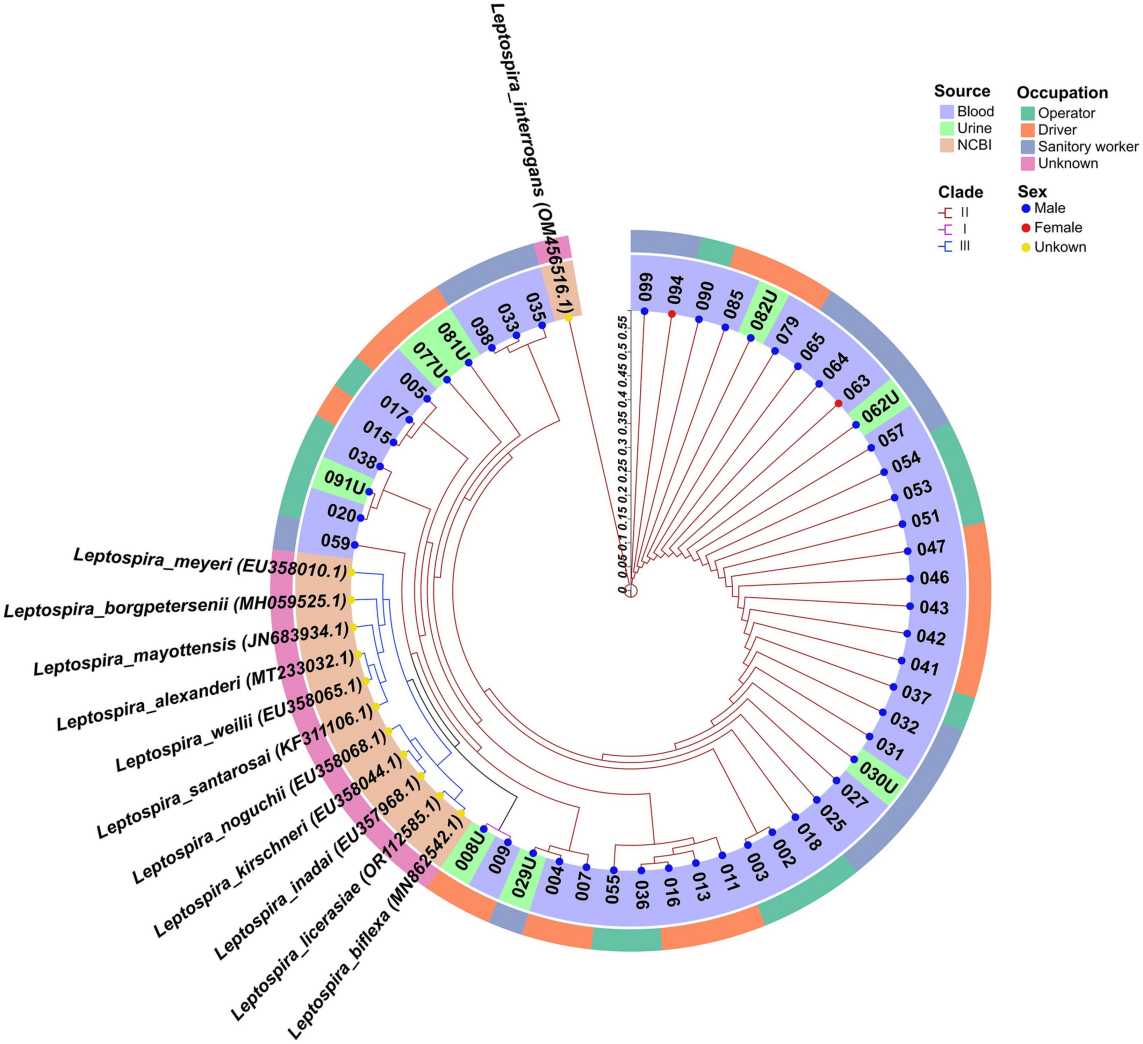
The neighbor-joining phylogenetic tree of the 50 sequences and 12 reference sequences based on *secY* gene retrieved from NCBI. The tree was generated together with MEGA X software and ChiPlot (www.chiplot.online) employing 1,000 bootstrap replicates. The three primary phylogenetic clades are labeled I to III and are color-coded in magenta, brown, and blue, respectively. The outer circle denotes the occupation of respondents, categorized as operator, driver, sanitary worker and unknown. The inner circle indicates the sample source (whole blood, urine and NCBI). Additionally, the colored points represent the sex of the individuals: blue for male, red for female, and yellow for unknown. Accession number of Genbank of each sequences were shown in the brackets.

DNA sequencing was performed on the amplicons of the *secY* gene. The phylogenetic tree, constructed using the Neighbor-Joining method, demonstrated that 2024HWGR008U and 2024HWGR009 were grouped in clade I, exhibiting only 84.0 and 84.1% homology with *L. interrogans* (GenBank accession number: OM56516.1) by DNASTAR MegAlign, respectively. The majority of sequences including from 7 urine samples and 41 blood samples clustered in clade II with *L. interrogans*, a known pathogenic bacterium, indicating a potentially close relationship with *L. interrogans*. The reference sequences retrieved from NCBI were all positioned in clade III (Figure 1).

### 3.4 Serology results

**Figure 2:**
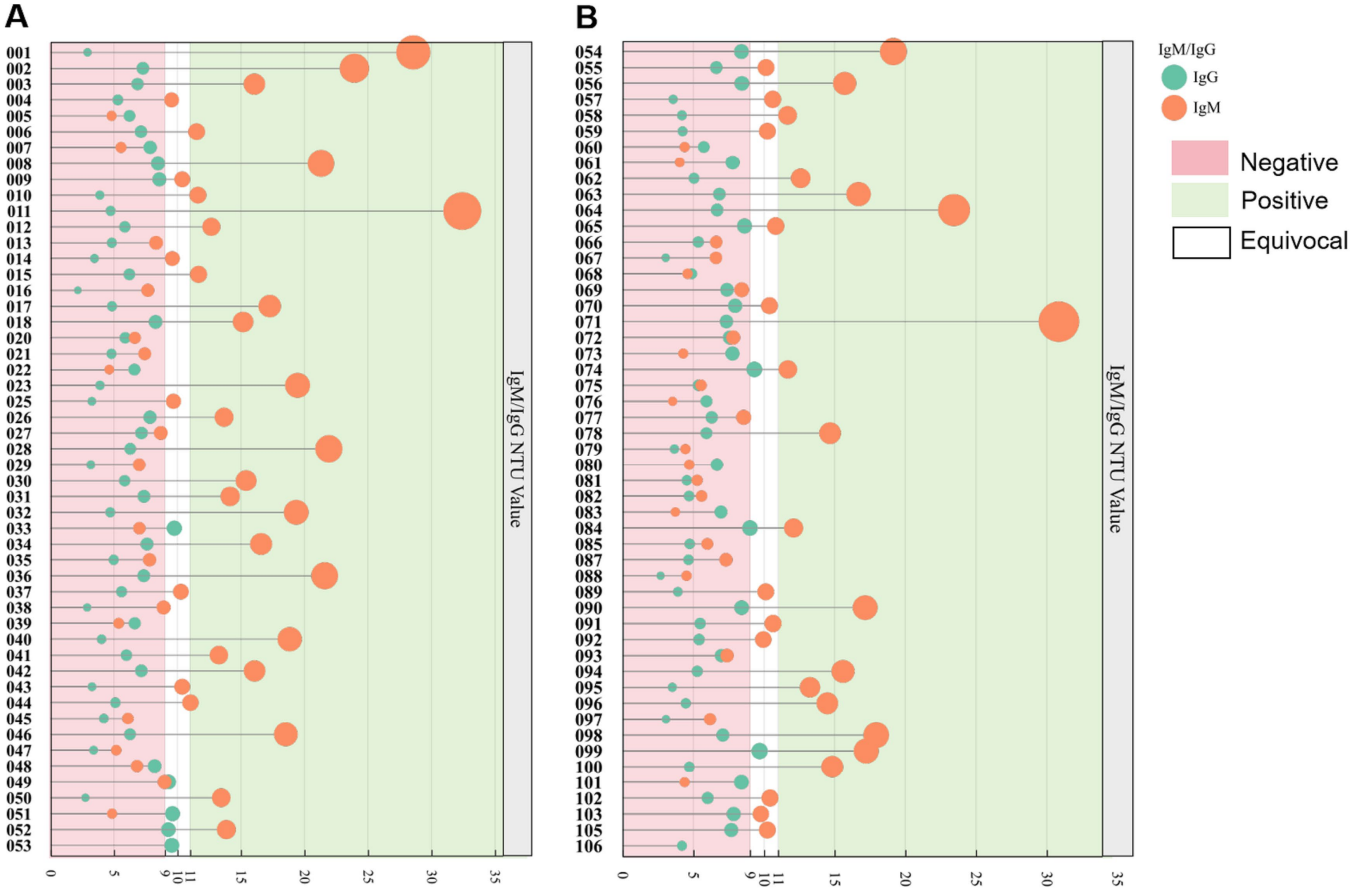
Evaluation of commercial *Leptospira* ELISA for IgM and IgG antibodies using 102 serum samples obtained from sanitation workers. **(A)** Serological outcomes for samples coded HWGR001-053. **(B)** Results for samples coded HWGR054-106. The green and orange markers denote IgM and IgG results, respectively. A value exceeding 11 NTU, indicated by a green background, is considered positive for IgM/IgG. Values ranging from 9 to 11 NTU, represented by a blank background, are classified as equivocal for IgM/IgG. Values below 9 NTU, shown with a pink background, are interpreted as negative for IgM/IgG.

In our study of 102 serum samples, the positive IgM rate was determined to be 43.1%, among which were 37 male and 7 female respondents. The IgM positivity rates observed among drivers, operators, and sanitary workers were 37.8, 54.5, and 41.7%, respectively. Regarding gender differences, the positivity rates were 40.7% for males and 63.6% for females (Table 4). Interestingly, none of the participants tested positive for IgG antibodies (Figure 2).

**Table 4:** Display of the distribution and prevalence of *Leptospira* in biosamples collected.

Features	IgM Positivity rate (%) [n^a^/N]	IgM Equivocal rate (%) [n^b^/N]	IgG Positivity rate (%) [n^c^/N]	*secY* gene detected in whole blood samples		*secY* gene detected in urine samples	
				Positivity rate (qPCR) [n^d^/N]	Positivity rate (PCR) [n^d*^/N]	Positivity rate (qPCR)[n^e^/N]	Positivity rate (PCR) [n^e*^/N]
Male	40.7% [37/91]	16.5% [15/91]	0	34.1% [31/91]	44.0% [40/91]	8.8% [8/91]	8.8% [8/91]
Female	63.6% [7/11]	18.2% [2/11]	0	18.2% [2/11]	18.2% [2/11]	0	0
Total	43.1% [44/102]	16.7% [17/102]	0	32.4% [33/102]	41.2% [42/102]	7.8% [8/102]	7.8% [8/102]
Drivers	37.8% [14/37]	10.8% [4/37]	0	29.7% [11/37]	40.5% [15/37]	10.8% [4/37]	10.8% [4/37]
Operators	54.5% [12/22]	18.1% [4/22]	0	45.5% [10/22]	59.1% [13/22]	4.5% [1/22]	4.5% [1/22]
Sanitary workers	41.7% [18/43]	20.9% [9/43]	0	27.9% [12/43]	32.6% [14/43]	7.0% [3/43]	7.0% [3/43]

N represents the number of different features. n^a^ and n^c^ indicate the positive counts of IgM and IgG, n^b^ represent the equivocal counts. n^d^ and n^e^ denote the positive counts of the secY gene found by qPCR method in whole blood and urine samples, n^d*^ and n^e*^ denote the positive counts of the secY gene found by conventional PCR method in whole blood and urine samples, respectively. PCR and qPCR in brackets represent the outcomes were applied by qPCR and conventional PCR methods.

### 3.5 Bacterial culture

Samples that tested positive by PCR and qPCR were subsequently cultured. The results showed that whole blood samples and urine samples molecular tested positive were determined to be culture-negative after 2 months’ observation.

## 4 Discussion

*Leptospira* are the causative agents of leptospirosis. The clinical manifestation of the infection varies depending on the host and the infecting serovar, ranging from an asymptomatic carrier state to severe, acute disease such as inflenza-like headaches, hepatic and renal failure, lung haemorrhage and death (18). The mechanisms underlying the manifestation of disease symptoms in the acute versus carrier states of leptospirosis are evidently distinct. However, research has predominantly concentrated on the acute form of the disease, particularly as it presents in humans. *Leptospira* can colonize the renal tubules of carrier hosts like rodents, cattle, pigs, and dogs, and are excreted in urine. Some hosts show no symptoms, indicating a long-term evolutionary relationship with *Leptospira* (34). In our current study we found that all respondents tested positive (PCR or IgM) had no symptoms like fever, cough or headache. None of them had vaccined before. 8 respondents who had leptospirosis were all male which indicated male may be more susceptible to female. This observation is supported by various studies that highlight the differences in immune response and disease outcomes between sexes. A study involving male and female hamsters demonstrated that male hamsters were significantly more susceptible to lethal infections with lower doses of pathogenic *Leptospira* than their female counterparts, with only 6.3% of males surviving the infection compared to 68.7% of females (35). The underlying mechanisms for this disparity may involve biological and physiological differences. The outer membrane lipoprotein LipL32 is exclusively present in pathogenic *Leptospira* species (36, 37) and represents the most abundant surface membrane protein (38). qPCR analysis targeting the *lipL32* gene yielded no amplicons. In contrast, the *secY* gene produced an amplicon corresponding to the expected band size of 285 bp. Both methods detected 27 blood samples, whereas all urine samples tested positive by qPCR and were capable of producing 8 amplicons. This finding suggests that blood samples may present a greater complexity than urine samples during the extraction process. Our data revealed that the ability of *Leptospira* lack of virulence to persist in the host, even at lower concentrations indicated by higher *Ct* values aligns with findings that suggest a nuanced interaction between *Leptospira* and its host, particularly in the context of immune responses and colonization dynamics (39). It is hypothesized that the colonization may have occurred silently with no symptoms in a highly insidious manner. Notably, a transposon mutant lacking the *lipL32* gene demonstrated neither a reduction in virulence in animal models nor any impact on interactions with host molecules *in vitro*, as reported in previous studies (40). The function of the major outer membrane protein LipL32 remains elusive; however, elucidating its role is crucial given its status as one of the most prevalent outer membrane proteins in the pathogenic *Leptospira* repertoire. Studies have shown that the innate immune responses in different host species can vary significantly, influencing the degree of activation of immune cells such as polymorphonuclear cells in cattle, which are natural reservoir hosts for *Leptospira* (41). A study (34) found that a dose of 10^5^ leptospires was optimal for mice, as it led to survival and renal colonization without disease symptoms or detectable leptospires in the blood. Two other studies tested mutants in colonization, revealing that Lip32L was unnecessary for rat kidney colonization (40). This suggests that other factors or proteins may compensate for the absence of Lip32L, allowing the bacteria to establish themselves within the host environment. It may explained that *lip32L* were not detectable in blood or urine samples from sanitation workers. The results was consistent to other study which note that no leptospiral virulence factors have been identified in the context of host colonization (42).

Serological analyses revealed that the respondents had very recent infections, with the majority exhibiting no prior infection history or clinical symptoms. Our data indicated that the IgM positivity rate among operators was higher compared to the other two occupational groups. Additionally, absolute count of IgM-positive cases was higher in males than in females. This observed disparity may be attributable to the smaller size of the female cohort within our study population, or potentially reflect inherent sex-based differences in exposure or immune response. Notably, a similar pattern of male predominance was reported in a descriptive cross-sectional study from Paraguay involving 339 workers from the Department of Urban Cleanliness of Asuncion, which found a leptospirosis seroprevalence of 8.6% (29/339), with all seropositive cases being male (43). This findings aligns with the result of a hospital-based study conducted among febrile cases in northeastern Malaysia, highlighting the high-risk occupational groups, predominately male who are at greater risk due to occupational exposure and other factors (44). A study of 303 urban sanitation workers in Kota Kinabalu, Sabah, reported a MAT seropositivity rate of 43.8% (133/303). Among these seropositive individuals, 29 (21.8%) were PCR-positive (45), a finding consistent with our results. Subsequent nucleotide sequencing confirmed *Leptospira* presence, and phylogenetic analysis identified the strains as belonging to the pathogenic group. Separately, a study of patients with acute febrile illness found that 119 out of 811 (14.7%) tested positive by *Leptospira* IgM ELISA (46). This high proportion of seropositivity and PCR-positive cases indicates a substantial risk of asymptomatic leptospirosis among urban sanitation workers. Besides, IgM positivity suggests recent exposure to *Leptospira*, it does not necessarily indicate progression to symptomatic disease. Some individuals may clear the infection without developing symptoms, whereas others may progress to symptomatic illness. IgM positivity suggests recent exposure to *Leptospira*, it does not necessarily indicate progression to symptomatic disease. Some individuals may clear the infection without developing symptoms, whereas others may progress to symptomatic illness (21, 47). Several factors can influence the IgG response in asymptomatic cases. In the context of *Leptospira*, the virulence of the infecting strain may be a contributing factor. For instance, studies in mice have demonstrated that more virulent strains of *L. interrogans* induce higher levels of IgG subclasses (48), suggesting that strains lacking virulence may elicit reduced or negligible IgG responses. Further experiments are necessary to elucidate the mechanisms underlying this phenomenon.

Culturing pure *Leptospira* is difficult due to its slow growth and vulnerability to contamination, which often results in mixed bacterial morphologies (15). Contamination hinders *Leptospira* growth, necessitating an extra filtration step that can reduce the number of bacteria. In our study, we failed to culture it, possibly because urine and blood samples were not processed within 24 h. For urine samples specifically, this duration within the acidic urinary environment poses a significant challenge. *Leptospira* are highly sensitive to low pH, which can rapidly impact their viability and ability to survive transport. Prolonged exposure to acidic urine during transport likely severely compromised the viability of any *Leptospira* present before culture initiation. Secondly it may due to the extremely low concentration in those samples. Some researchers (49) suggest adding sulphamethoxazole, trimethoprim, amphotericin B, fosfomycin, and fluorouracil to the medium to eliminate contaminants and improve selectivity.

Sanitation workers, such as those handling waste collection, transport, and sorting, face an increased risk of leptospirosis due to their constant exposure to contaminated settings. Our study shows that extended work hours and jobs in waste disposal elevate infection risk, whereas wearing boots and masks properly and cleaning with soap can greatly reduce this risk. Multiple studies (50, 51) have shown that using PPE and maintaining good personal hygiene are essential for lowering exposure to *Leptospira* and decreasing infection risk.

Our study is subject to several limitations. Firstly, the relatively small sample size may have diminished the statistical power necessary to detect significant differences between groups, thereby increasing the likelihood of Type II errors. A notable methodological limitation is the absence of a dedicated control group; the study focused solely on sanitation workers without recruiting a comparable cohort of non-sanitation workers from the same geographical area. Furthermore, the study could have benefited from the application of additional methods, such as MAT, ddPCR, and mNGS, to enhance the detection and serotyping of *Leptospira*. We plan to recruit additional participants across multiple waste transfer stations to enhance longitudinal surveillance of IgM and IgG serological profiles. This expanded cohort will provide deeper insights into antibody response dynamics and infection trends over time.

## 5 Conclusion

Asymptomatic carriers may pose a greater risk than symptomatic patients. Despite the absence of clinical symptoms, there remains a potential risk of transmission. The colonization of *Leptospira* in host organisms involves complex mechanisms that warrant further investigation. Investigating these interactions could provide insights into how *Leptospira* successfully colonize and persist in their hosts, despite the immune challenges they face. We recommend that routine surveillance should include the detection of the *secY* gene, in addition to the commonly targeted *lipL32* gene, an important virulence factor. In Future research should focus on identifying and characterizing these alternative mechanisms to enhance our understanding of leptospiral pathogenesis and improve therapeutic interventions. Sanitation workers are at significant risk of *Leptospira* infection. It is imperative for these workers to possess knowledge about infection risks and preventive measures to mitigate the likelihood of infection.

## Data Availability

The authors declare that data supporting the findings of this study is available within the Supplementary material. Any raw data files are available from the corresponding author upon reasonable request.

## Footnotes

^1^ www.chiplot.online
