# Supplementary material for: Unveiling Leptospira prevalence and exposure in sanitation workers, a cross-sectional study in Ningbo City, China
Source: Front Public Health. 2025 Jul 2;13:1627155. doi: 10.3389/fpubh.2025.1627155 (PMC12263941; doi:10.3389/fpubh.2025.1627155)
Supplement: Supplementary file 1 [file Table_1.docx]

**Supplementary TABLE 1 Positive results of PCR assays from whole blood samples**

| **Sample code** | **Sex** | **Age** | **Occupation** | ***secY* gene (qPCR assays)** | ***lipL32* gene (qPCR assays)** | ***Ct* value** | ***secY* gene (PCR assays)** | **Homology with *L. interrogans(%)*** |
| --- | --- | --- | --- | --- | --- | --- | --- | --- |
| **2024HWGR002** | Male | 57 | Operator | ＋ | - | 34.57 | ＋ | 90.5% |
| **2024HWGR003** | Male | 57 | Operator | ＋ | - | 35.2 | ＋ | 98.7% |
| **2024HWGR004** | Male | 52 | Driver | ＋ | - | 33.9 | ＋ | 84.7% |
| **2024HWGR005** | Male | 50 | Driver | - | - | / | + | 92.4% |
| **2024HWGR006** | Male | 49 | Driver | ＋ | - | 32.87 | - | / |
| **2024HWGR007** | Male | 53 | Driver | ＋ | - | 34.82 | ＋ | 91.2% |
| **2024HWGR009** | Male | 58 | Driver | ＋ | - | 34.98 | ＋ | 84.1% |
| **2024HWGR011** | Male | 52 | Driver | ＋ | - | 34.77 | ＋ | 97.0% |
| **2024HWGR012** | Male | 54 | Driver | ＋ | - | 35.15 | - | / |
| **2024HWGR013** | Male | 54 | Driver | ＋ | - | 35.33 | ＋ | 98.0% |
| **2024HWGR015** | Male | 40 | Driver | ＋ | - | 35.58 | ＋ | 96.6% |
| **2024HWGR016** | Male | 60 | Driver | ＋ | - | 33.73 | ＋ | 92.5% |
| **2024HWGR017** | Male | 50 | Operator | ＋ | - | 33.72 | ＋ | 97.6% |
| **2024HWGR018** | Male | 48 | Operator | ＋ | - | 33.6 | ＋ | 97.2% |
| **2024HWGR020** | Male | 50 | Operator | ＋ | - | 34.78 | ＋ | 96.1% |
| **2024HWGR025** | Male | 53 | Sanitary worker | ＋ | - | 34.71 | ＋ | 99.6% |
| **2024HWGR027** | Male | 57 | Sanitary worker | ＋ | - | 34.33 | ＋ | 98.8% |
| **2024HWGR029** | Male | 59 | Sanitary worker | ＋ | - | 35.53 | - | / |
| **2024HWGR031** | Male | 58 | Sanitary worker | ＋ | - | 34.4 | ＋ | 99.2% |
| **2024HWGR032** | Male | 56 | Sanitary worker | ＋ | - | 36.65 | ＋ | 95.7% |
| **2024HWGR033** | Male | 56 | Sanitary worker | ＋ | - | 34.73 | ＋ | 98.6% |
| **2024HWGR035** | Male | 57 | Sanitary worker | ＋ | - | 35.44 | ＋ | 96.2% |
| **2024HWGR036** | Male | 45 | Operator | ＋ | - | 34.63 | ＋ | 94.9% |
| **2024HWGR037** | Male | 62 | Operator | ＋ | - | 35.4 | ＋ | 98.8% |
| **2024HWGR038** | Male | 33 | Operator | ＋ | - | 35.01 | ＋ | 97.2% |
| **2024HWGR041** | Male | 55 | Driver | ＋ | - | 32.5 | ＋ | 99.2% |
| **2024HWGR042** | Male | 53 | Driver | ＋ | - | 35.22 | + | 98.8% |
| **2024HWGR043** | Male | 48 | Driver | - | - | / | + | 98.8% |
| **2024HWGR046** | Male | 44 | Driver | - | - | / | + | 92.5% |
| **2024HWGR047** | Male | 49 | Driver | - | - | / | + | 96.2% |
| **2024HWGR051** | Male | 64 | Operator | - | - | / | + | 97.4% |
| **2024HWGR053** | Male | 56 | Operator | - | - | / | + | 99.2% |
| **2024HWGR054** | Male | 52 | Operator | - | - | / | + | 100% |
| **2024HWGR055** | Male | 49 | Operator | - | - | / | + | 94.7% |
| **2024HWGR057** | Male | 51 | Sanitary worker | ＋ | - | 34.44 | ＋ | 98.4% |
| **2024HWGR059** | Male | 55 | Sanitary worker | - | - | / | + | 85.1% |
| **2024HWGR063** | Female | 46 | Sanitary worker | ＋ | - | 35.17 | ＋ | 99.2% |
| **2024HWGR064** | Male | 56 | Sanitary worker | - | - | / | + | 96.4% |
| **2024HWGR065** | Male | 54 | Sanitary worker | ＋ | - | 35.57 | ＋ | 100% |
| **2024HWGR072** | Male | 60 | Operator | ＋ | - | 33.39 | - | / |
| **2024HWGR079** | Male | 51 | Driver | - | - | / | + | 99.6% |
| **2024HWGR085** | Male | 52 | Driver | - | - | / | + | 97.2% |
| **2024HWGR089** | Male | 58 | Operator | ＋ | - | 20.74 | - | / |
| **2024HWGR090** | Male | 58 | Operator | - | - | / | + | 100% |
| **2024HWGR094** | Female | 48 | Sanitary worker | ＋ | - | 29.6 | ＋ | 100% |
| **2024HWGR098** | Male | 54 | Sanitary worker | - | - | / | + | 98.8% |
| **2024HWGR099** | Male | 47 | Sanitary worker | - | - | / | + | 99.6% |
| **2024HWGR100** | Male | 55 | Sanitary worker | ＋ | - | 33.71 | - | / |

qPCR assays and PCR assays in brackets represent the outcomes were applied by qPCR and conventional PCR methods.

U denotes that the samples were urine. qPCR asssays and PCR assays in brackets represent the outcomes were applied by qPCR and conventional PCR methods.
