# Supplementary material for: Unveiling Leptospira prevalence and exposure in sanitation workers, a cross-sectional study in Ningbo City, China
Source: Front Public Health. 2025 Jul 2;13:1627155. doi: 10.3389/fpubh.2025.1627155 (PMC12263941; doi:10.3389/fpubh.2025.1627155)
Supplement: Supplementary file 2 [file Table_2.docx]

**Supplementary TABLE 2 Positive results of PCR assays from urine samples**

| **Sample code** | ***secY* gene (qPCR assays)** | ***lipL32* gene (qPCR assays)** | ***Ct value*** | ***secY* gene (PCR assays)** | **Homology with *L. interrogans(%)*** |
| --- | --- | --- | --- | --- | --- |
| **2024HWGR008U** | ＋ | - | 34.74 | ＋ | 84.0% |
| **2024HWGR029U** | ＋ | - | 35.26 | ＋ | 91.1% |
| **2024HWGR030U** | ＋ | - | 36.72 | ＋ | 97.2% |
| **2024HWGR062U** | ＋ | - | 32.15 | ＋ | 97.2% |
| **2024HWGR077U** | ＋ | - | 34.28 | ＋ | 92.1% |
| **2024HWGR081U** | ＋ | - | 33.61 | ＋ | 92.5% |
| **2024HWGR082U** | ＋ | - | 31.82 | ＋ | 98.4% |
| **2024HWGR091U** | ＋ | - | 36.93 | ＋ | 94.2% |
